# A Decade Later: Trends in Fellowship Training and Secondary Board Certification Among Emergency Medicine Residency Leaders

**DOI:** 10.5811/westjem.54378

**Published:** 2026-09-09

**Authors:** Danielle Langan, Lindsay Shogan, Shorok E. Hassan, William Caputo, Abbas Husain, Jerel Chacko, Amin Mohamadi, Josh Greenstein, Barry Hahn

**Affiliations:** * Northwell Health, New Hyde Park, New York; † Staten Island University Hospital, Northwell Health, Department of Emergency Medicine, Staten Island, New York; ‡ Hadassah University Medical Center–Ein Kerem, Department of Emergency Medicine, Jerusalem, Israel

## Abstract

**Introduction:**

Residency program leaders shape the academic pipeline in emergency medicine (EM). We assessed current demographics and postgraduate training among EM residency leadership in the United States.

**Methods:**

We conducted a national, cross-sectional survey of all Accreditation Council for Graduate Medical Education–accredited EM residency program leadership via an online, confidential questionnaire. Primary outcomes were the prevalence of any postgraduate fellowship training and any secondary board certification (ie, additional board certification beyond EM) as a complementary marker of advanced credentialing distinct from fellowship training. Programs were grouped by National Resident Matching Program (NRMP) region.

**Results:**

We received 103 program responses (35.9%). Any fellowship training was reported by 25.5% of program directors (PD), 40.0% of associate program directors (APD), and 42.8% of assistant PDs. Compared with the 2014–2015 national survey, fellowship training increased from approximately 20–25% to 40–43% among APD and assistant PDs, while remaining similar among PDs (~25% previously vs 26% currently). Any secondary board certification was reported by 8.8% of PDs, 5.0% of APDs, and 8.2% of assistant PDs. Medical education was the most common fellowship (*n* = 54), followed by ultrasound (*n* = 24), toxicology (*n* = 16), simulation (*n* = 15), pediatric EM (*n* = 11), critical care (*n* = 7), emergency medical services (*n* = 5), and administration (*n* = 3). Programs in the western United States reported the highest proportions of leaders with either fellowship or secondary certification across roles.

**Conclusion:**

Postgraduate fellowship and secondary board certification remain uncommon overall among EM residency leaders, but fellowship completion is more prevalent among junior leaders compared with a decade earlier. These data provide an updated, role-specific snapshot to inform leadership development and workforce planning.

## INTRODUCTION

Residency leaders in emergency medicine (EM), including program directors (PD), associate PDs (APD), and assistant PDs, shape curricula, recruitment, mentorship, and scholarly priorities for the specialty’s future workforce. Interest in postgraduate fellowship training has expanded over time, but peer-reviewed data about how such training is represented among EM residency leadership are limited. In a prior national survey (2014–2015) of Accreditation Council for Graduate Medical Education (ACGME)–accredited EM programs, –fellowship training among residency leaders was relatively uncommon (PDs, 21.4%; APDs, 20.3%; assistant PDs, 24.9%), and secondary board certification was even less prevalent. Regional variation was observed, with the Western United States reporting the highest proportion of additional training.

Over the same period, the landscape of EM training has expanded substantially. Between 2005 and 2019, the number of accredited EM residency programs approximately doubled, and more recent national reports document continued growth. In parallel, the breadth and availability of EM fellowships and subspecialty pathways have expanded, including the emergence of additional accredited subspecialty–training options. These changes have practical implications for interpreting our findings: Junior faculty entering leadership roles today are more likely to have trained during an era with greater fellowship availability, and the decision to pursue fellowship also carries a meaningful opportunity cost relative to entering independent practice earlier.

Concurrently, literature from other specialties suggests that advanced training and credentialing are common among faculty in leadership roles, although patterns vary by field and are associated with persistent demographic disparities. Because fellowship training is not universally required in EM, we cite these cross-specialty studies primarily to illustrate how additional training and credentialing can shape leadership pipelines, rather than to imply equivalence in baseline fellowship expectations across fields. For example, ophthalmology leadership and fellowship directors are frequently subspecialty–trained, with studies documenting high rates of fellowship training among leaders across organizations and programs.^,^ Given ongoing interest in leadership representation, we also describe sexdistribution across EM residency leadership roles.^,^

It remains unclear whether the training profile of EM residency leadership has shifted over the last decade. It is unclear whether fellowship completion and secondary board certification have become more prevalent, whether regional differences persist, and whether the earlier finding that assistant PDs with shorter leadership tenure are more likely to report fellowship training reflects a generational shift in leadership qualifications. The objective of this study was to describe current demographics, fellowship training, and secondary board certification among EM residency leaders in the US and to compare these patterns with those reported a decade earlier, including variation by leadership role and geographic region.

**Population Health Research Capsule**

What do we already know about this issue?

*Fellowship training was previously uncommon among emergency medicine (EM) residency leaders*.

What was the research question?

*How common are fellowship training and secondary board certification among EM residency leaders?*

What was the major finding of the study?

*Fellowship training was reported by 25.5% of program directors (PDs), 40.0% of associate PDs, and 42.8% of assistant PDs*.

How does this improve population health?

*These data inform EM leadership development, mentorship, and workforce planning across training programs*.

## METHODS

### Design and Setting

We conducted a national, cross-sectional survey of all ACGME–accredited EM residency programs in the United States. The sampling included each categorical EM residency identified through Emergency Medical Residents Association Match and program/department websites at the time of survey launch. Only ACGME–accredited programs were included (*N* = 287). Eligible respondents were PDs, APDs, assistant PDs, or a designated program administrator who could report leadership characteristics for their program. Length of time in residency leadership reflected cumulative tenure in leadership roles regardless of whether service occurred at a single or multiple institutions. This study received an exempt determination from the Northwell Health Institutional Review Board.

### Recruitment and Survey Administration

An online, confidential questionnaire was distributed to program leadership via the Council of Emergency Medicine Residency Directors (CORD) listserv and direct program emails during February–April 2025. The initial invitation was sent in February 2025, with follow-up reminders through April 2025. Nonrespondents received up to two reminder emails at approximately two-week intervals. The survey instrument was identical to the one used in the prior 2014–2015 study to enable direct comparison across time. Before survey closure, programs that had not responded were contacted individually to encourage participation.

### CHERRIES Checklist Reporting (Internet Survey Specifics)

This was an internet-based survey distributed via the CORD listserv and direct program emails during February–April 2025, with up to two reminders sent at two-week intervals to nonrespondents. Participation was voluntary with implied consent by completion. We requested one response per program, with preference for the PD. If duplicate program responses were received, we retained the most complete or, when equal, the earliest complete record. No personal identifiers were collected. Data were stored in institutional systems and then exported to Microsoft Excel (Microsoft Corporation, Redmond, WA) for analysis. The overall response rate was 35.9% (103/287), and the survey completion rate was 35.2% (101/287). Among those who responded, 98.1% (101/103) completed the section on sex. Responding programs were similar to the national distribution of ACGME EM programs by National Resident Matching Program (NRMP) region and program length. Percentages were computed using nonmissing denominators. We did not use view-rate metrics such as email opens, IP- or cookie-checks, or incentives. See Appendix 1 (CHERRIES checklist) for itemized reporting.

### Measures

Our primary outcome measure was the prevalence of any postgraduate fellowship training among EM residency leaders. Secondary outcome measures included the prevalence of secondary board certification, differences in training patterns by leadership role, regional variation by NRMP region, and the distribution of reported fellowship types. For each leadership role, respondents reported sex, combined years in residency leadership, completion of any postgraduate fellowship, and any secondary board certification. Because leadership structures vary across institutions, APD and assistant PD titles are not standardized nationally; we therefore report role-specific findings as described by each program. The survey collected data for one PD, up to two APDs, and up to four assistant PDs per program, consistent with the prior instrument. If a program had more individuals in a role than the survey allowed, the designated respondent reported the individuals most directly involved in residency leadership, and each individual was counted only once.

The following operational definitions were used for training and credentialing: fellowship training, completion of a postgraduate fellowship after residency; and secondary board certification, any additional board certification beyond EM. For pediatric pathways, a pediatric EM fellowship paired with pediatrics board certification was considered fellowship training and not dual-counted as secondary board certification.

Programs were assigned to NRMP regions (Western, Northeastern, Central, Southern). A priori, pediatric EM fellowships with pediatrics board certification were classified as fellowships (not dual-counted as secondary certification) for consistency. The complete survey instrument and response options appear in Appendix 2.

### Data Management and Analysis

We aggregated responses at the program level and calculated descriptive statistics. Prespecified sensitivity analyses to test robustness included (1) excluding programs with incomplete role reporting and (2) reclassifying programs requiring a. pediatric EM fellowship and pediatrics board certification as requiring both a fellowship and a secondary board certification to test robustness. Analyses were performed in Microsoft Excel.

## RESULTS

Surveys were sent to all ACGME–accredited EM residency programs (*N* = 287). We received 103 program responses (35.9%), of which 101 were complete (35.2%). Regional response rates were as follows: Northeast, 52.4% (43/82); Central, 23.6% (17/72); South, 31.9% (30/94); and West, 30.8% (12/39). One responding program did not report a region. These produced 410 role-level entries, including 102 PDs, 100 APDs, and 208 assistant PDs. Respondents represented all NRMP regions and both three-year and four-year program lengths (Table 1). Additional descriptors are provided in Table 2.

The prevalence of any postgraduate fellowship training was 25.5% among PDs (26/102), 40.0% among APDs (40/100), and 42.8% among assistant PDs (89/208). The prevalence of any secondary board certification was 8.8% among PDs (9/102), 5.0% among APDs (5/100), and 8.2% among assistant PDs (17/208) (Figure 1).

Across all leaders, reported fellowships included medical education (54/410, 13.2%), ultrasound (24/410, 5.9%), toxicology (16/410, 3.9%), simulation (15/410, 3.7%), pediatric EM (11/410, 2.7%), critical care (7/410, 1.7%), emergency medical services (5/410, 1.2%), and administration (3/410, 0.7%). An additional 20 leaders (20/410, 4.9%) reported “Other” fellowship types. No fellowship was reported for 255 leaders (255/410, 62.2%).

Secondary board certifications included internal medicine (9/410, 2.2%), pediatrics (6/410, 1.5%), critical care (5/410, 1.2%), and other specialties (11/410, 2.7%), while no secondary board certification was reported for 379 leaders (379/410, 92.4%).

The proportion identifying as female was 37.3% among PDs (38/102), 42.0% among APDs (42/100), and 50.5% among assistant PDs (105/208). The proportion identifying as male was 61.8% among PDs (63/102), 57.0% among APDs (57/100), and 46.6% among assistant PDs (97/208). “Prefer not to answer” responses accounted for 1.0% of PDs (1/102), 1.0% of APDs (1/100), and 1.9% of assistant PDs (4/208), with missing sex data for 1.0% of assistant PDs (2/208).

Cumulative time in residency leadership was as follows: < 5 years for 28.4% of PDs (29/102), 28.0% of APDs (28/100), and 65.9% of assistant PDs (137/208); 5–10 years for 34.3% of PDs (35/102), 53.0% of APDs (53/100), and 26.9% of assistant PDs (56/208); and > 10 years for 36.3% of PDs (37/102), 18.0% of APDs (18/100), and 6.7% of assistant PDs (14/208).

For the combined endpoint of either fellowship training or secondary board certification, proportions by NRMP region and leadership role are shown in Figure 2. In the Western region, 41.7% of PDs (5/12), 60.0% of APDs (6/10), and 51.9% of Asst PDs (14/27) reported advanced training. In the Northeastern region, the corresponding proportions were 34.9% (15/43), 42.1% (16/38), and 47.8% (33/69). In the Central region, 17.6% of PDs (3/17), 52.9% of APDs (9/17), and 48.8% of Asst PDs (20/41) reported advanced training. In the Southern region, the proportions were 26.7% (4/15), 26.7% (4/15), and 33.3% (22/66), respectively.

## DISCUSSION

This national, cross-sectional survey of ACGME-accredited EM residency programs in the US provides an updated assessment of leadership demographics and postgraduate training a decade after the original analysis.^1^ Surveys were distributed to 287 programs, with 103 responses (35.9%), of which 101 were complete (35.2%). Although the response rate was lower than that of the prior study, the absolute number of programs represented remains substantial and includes broad geographic and institutional diversity.^,^ In the 2014–2015 survey, responses were obtained from 145 of 164 ACGME–accredited EM programs (88%).

Compared with the prior national survey conducted in 2014–2015, fellowship training among residency leaders has increased most notably among faculty in junior leadership roles. In the earlier study, fellowship completion was reported by 21.4% of PDs, 20.3% of APDs, and 24.9% of assistant PDs. In the current study, corresponding rates were 25.5% for PDs, 40.0% for APDs, and 42.8% for assistant PDs, representing an absolute increase of approximately 15–18 percentage points among associate and assistant PDs. The distribution of fellowship types has also changed, with medical education now the most frequently reported fellowship, whereas toxicology was previously the most frequent. Regional differences persist, with Western region programs continuing to report higher proportions of leaders with advanced training across roles. Secondary board certification remains uncommon across leadership positions in both study periods.

These findings suggest that fellowship training remains relatively uncommon among senior leaders but is more frequently represented in junior leadership roles, supporting a possible shift in the leadership pipeline over time.

Prior work examining outcomes among EM residency graduates in the US found that pursuing postgraduate fellowship training was uncommon overall, with approximately 4–9% of graduates entering fellowship, depending on residency format. In this context, the fellowship completion rates observed among residency leaders in the present study, particularly among APDs and assistant PDs, are substantially higher than historical baseline rates among EM graduates. This suggests that residency leadership represents a selected subgroup with greater engagement in formal postgraduate training than the broader EM workforce.

Secondary board certification was relatively uncommon, reported by fewer than 10% of respondents across all leadership roles. Pediatric board certification, often paired with a pediatric EM fellowship, was the most frequent category. The overall low prevalence of secondary certification mirrors the findings of the prior national survey, suggesting that this pathway remains a less common route to residency leadership.^1^

Sex distribution varied by leadership role, with greater representation of women in more junior roles (Table 1), consistent with broader trends in academic EM. Leadership tenure similarly differed by role, with PDs reporting longer time in position and assistant PDs more commonly earlier in their leadership trajectory (Table 1). This pattern suggests that fellowship training is more prevalent earlier in leadership roles and less common at the PD level.

We observed regional differences in fellowship training and secondary board certification. Program heads in We programs in the Western US reported the highest prevalence of fellowship or secondary board certification across all roles, followed by the Northeast and Central regions, with the Southern region reporting the lowest prevalence. These trends may reflect regional differences in academic infrastructure, fellowship availability, or institutional emphasis on postgraduate training.

Taken together, the findings in this study provide an updated, role-specific snapshot of qualifications within EM residency leadership that programs, departments, and trainees can use for workforce planning and mentorship. For programs recruiting future leaders, the pattern suggests growing value placed on structured postgraduate training, especially in education-facing domains, without implying it is universally required. For trainees aspiring to leadership, the data highlight common pathways and may help guide career planning.

Future work should investigate whether increased fellowship training among junior leaders ultimately results in higher fellowship rates among PDs. It should also examine whether similar patterns are present among practicing emergency physicians outside leadership roles and whether advanced training is associated with measurable program outcomes, such as educational quality, scholarly productivity, or trainee recruitment. Repeating this analysis in another decade, using consistent methods and stronger response strategies, will be essential to confirm these trends and refine the implications for EM leadership.

## LIMITATIONS

This study has several limitations. The first is the survey response rate. We received 103 responses from 287 ACGME–accredited EM residency programs (35.9%), with 101 complete submissions (35.2%). A response rate in this range raises the possibility of nonresponse bias if programs with particular characteristics (eg, greater emphasis on fellowship training, specific leadership structures, or certain regions) were more or less likely to participate. Although this risk cannot be excluded, similar response rates are common in national surveys of EM educators and residency leadership and have been used to describe workforce characteristics when methodological transparency is maintained.^-^ The absolute sample size is large and includes programs from all NRMP regions, as well as a mix of program lengths, sizes, and institution types, supporting descriptive generalizability. Key directional patterns replicate prior national observations (eg, higher fellowship prevalence among junior leaders and low overall rates of secondary board certification), which bolsters confidence that the principal signals are not artifacts of selective response. We do not make population-level causal claims, and our conclusions are framed accordingly. Ultimately, these analyses are descriptive rather than inferential. We do not draw causal conclusions, and our findings should be interpreted accordingly. As a preliminary nonresponse check, the respondent pool reflected all NRMP regions and both 36- and 48-month formats in proportions consistent with national profiles.

Second, as with any self-report survey, responses are subject to recall and classification error. To minimize this, we prespecified operational rules. For example, the pediatric EM fellowship paired with the pediatrics boards was counted only as a fellowship, rather than being dual counted. We also reported free-text “other” entries transparently, such as medical education, simulation, sports medicine, resuscitation, and informatics (see Table 1 footnote). Still, some respondents may have used “other” to describe non-ACGME educational experiences, potentially misrepresenting formal fellowship categories.

The third limitation is the cross-sectional design. We describe prevalence at a single point in time and cannot determine trajectories for individual leaders or programs. The higher fellowship rates among assistant PDs could reflect differences between training eras rather than progression within leadership roles. As fellowship opportunities have expanded over the past decade, more recent graduates may enter leadership positions with higher baseline rates of postgraduate training, which could amplify these apparent generational differences.

A fourth limitation is the grouping of programs into the four NRMP regions for comparability. This grouping may hide variation within regions. Programs also differ in the number of APD and assistant PDs, which changes role denominators and can affect role-specific estimates. Finally, the percentage of missing data was low but present (generally ≤ 2% across program-characteristic items; see Table 2). We calculated percentages using nonmissing denominators and reported missingness and “other” categories in table footnotes for transparency.

## CONCLUSION

In this national survey of ACGME–accredited emergency medicine residency programs, fellowship training was uncommon among program directors but more prevalent among junior residency leaders, while secondary board certification remained infrequent. These data update the 2014–2015 baseline and inform future work on how training pathways relate to leadership development and program outcomes.

## Supplementary Information



## Figures and Tables

**Figure 1:**
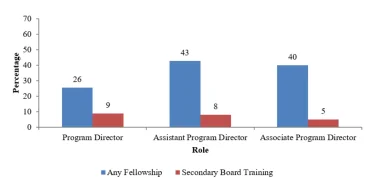
Prevalence of any fellowship training versus any secondary board certification by leadership role in a national survey of Accreditation Council for Graduate Medical Education-accredited emergency medicine residency programs in the United States.

**Figure 2:**
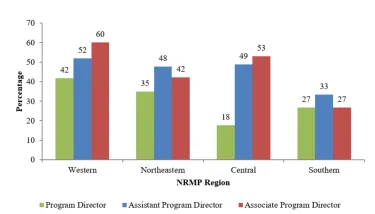
Prevalence of either fellowship training or secondary board certification by NRMP region and leadership role in a national survey of ACGME-accredited emergency medicine residency programs. *ACGME*, Accreditation Council for Graduate Medical Education; *NRMP*, National Resident Matching Program.

**Table 1:** Characteristics of emergency medicine leaders in a national survey of Accreditation Council for Graduate Medical Education–accredited programs.

	Program Director (*n* = 102)	Associate Program Director (*n* = 100)	Assistant Program Director (*n* = 208)	Total (*N* = 410)
Postgraduate Training and Board Certification				
Any fellowship training	26 (25.5%)	40 (40.0%)	89 (42.8%)	155 (37.8%)
Any secondary board certification	9 (8.8%)	5 (5.0%)	17 (8.2%)	31 (7.6%)
Fellowship Type				
Medical Education	12 (11.8%)	10 (10.0%)	32 (15.4%)	54 (13.2%)
Ultrasound	3 (2.9%)	10 (10.0%)	11 (5.3%)	24 (5.9%)
Toxicology	0 (0.0%)	4 (4.0%)	12 (5.8%)	16 (3.9%)
Simulation	1 (1.0%)	6 (6.0%)	8 (3.8%)	15 (3.7%)
Critical Care	2 (2.0%)	2 (2.0%)	3 (1.4%)	7 (1.7%)
Emergency Medical Services	2 (2.0%)	1 (1.0%)	2 (1.0%)	5 (1.2%)
Administration	0 (0.0%)	1 (1.0%)	2 (1.0%)	3 (0.7%)
Pediatric Emergency Medicine	1 (1.0%)	2 (2.0%)	8 (3.8%)	11 (2.7%)
Other*	5 (4.9%)	4 (4.0%)	11 (5.3%)	20 (4.9%)
Secondary Board				
Internal Medicine	5 (4.9%)	2 (2.0%)	2 (1.0%)	9 (2.2%)
Pediatrics	1 (1.0%)	0 (0.0%)	5 (2.4%)	6 (1.5%)
Critical Care	1 (1.0%)	1 (1.0%)	3 (1.4%)	5 (1.2%)
Other	2 (2.0%)	2 (2.0%)	7 (3.4%)	11 (2.7%)
Sex				
Female	38 (37.3%)	42 (42.0%)	105 (50.5%)	185 (45.1%)
Male	63 (61.8%)	57 (57.0%)	97 (46.6%)	217 (52.9%)
Prefer not to answer	1 (1.0%)	1 (1.0%)	4 (1.9%)	6 (1.5%)
Leadership Experience				
< 5 years	29 (28.4%)	28 (28.0%)	137 (65.9%)	194 (47.3%)
5–10 years	35 (34.3%)	53 (53.0%)	56 (26.9%)	144 (35.1%)
> 10 years	37 (36.3%)	18 (18.0%)	14 (6.7%)	69 (16.8%)

* Other includes free-text entries such as sports medicine, resuscitation, informatics, and non–ACGME training.

**Table 2:** Survey response metrics of Accreditation Council for Graduate Medical Education–accredited emergency medicine residency programs.

	Number of Respondents	Percentage (%)
Program Length		
3-year program	80	79.2
4-year program	21	20.8
Program Size (PGY-1 intake per year)		
Large (≥ 13 residents per year)	39	38.2
Medium (7–12 residents per year)	53	52
Small (1–6 residents per year)	10	9.8
Institution Type		
Academic medical center	51	50
Community hospital	14	13.7
County hospital	11	10.8
Hybrid (eg, community-academic affiliation)	26	25.5
Associate Program Directors per program		
0	26	25.5
1	52	51
2	24	23.5
Assistant Program Directors per program		
1	34	33.7
2	36	35.6
3	21	20.8
≥ 4	10	9.9

Percentages calculated with nonmissing denominators. Missing data included program length (1.9%), program size (1.0%), institution type (1.0%), associate program director count (1.0%), and assistant program director count (1.9%).

*PGY*, postgraduate year.
