# Supplementary material for: A Decade Later: Trends in Fellowship Training and Secondary Board Certification Among Emergency Medicine Residency Leaders
Source: West J Emerg Med. 2026 Sep 9;27(5):1243–9. doi: 10.5811/westjem.54378 (PMC13660578; doi:10.5811/westjem.54378)
Supplement: Supplementary file 1 [file wjem-27-1243-s001.pdf]

Appendix 1. CHERRIES Checklist (Internet Survey Reporting)

This appendix itemizes key elements of our internet-based survey in accordance with the CHERRIES framework and indicates where each item appears in the manuscript.

CHERRIES item	What we did	Where in manuscript
Survey design & mode	Internet-based, email/listserv distribution to EM residency leadership; voluntary; one response per program requested (program director preferred).	Methods: Recruitment and survey administration; CHERRIES reporting
Setting & field dates	[Month–Month, Year]; up to two reminders sent at approximately two-week intervals.	Methods: CHERRIES reporting
Participants & eligibility	All ACGME-accredited U.S. EM residency programs; respondent could be PD/APD/Asst PD or designated administrator able to report leadership characteristics.	Methods: Design and setting
Informed consent & IRB	Implied consent by completion; Institutional Review Board exempt determination (Northwell Health).	Methods: Design and setting
Duplicate prevention	Requested one response per program; if duplicate program responses were detected, retained the most complete (or earliest complete) record.	Methods: CHERRIES reporting
Survey instrument	Final instrument and response options provided verbatim in Appendix A (as administered).	Methods: Measures; Appendix A

Reminders	Up to two reminder emails sent at ~two-week intervals to non-respondents; near survey close, individual outreach to non-responding programs.	Methods: Recruitment and survey administration
Data protection & storage	No personal identifiers collected; data stored on institutional systems; exported to Microsoft Excel (Microsoft Corporation, Redmond, WA) for analysis.	Methods: CHERRIES reporting
Outcomes & denominators	Primary outcomes: any postgraduate fellowship; any secondary board certification. Percentages computed using non-missing denominators. Pediatric EM fellowship paired with Pediatrics boards counted as 'fellowship' only (not dual-counted).	Methods: Measures; Data management and analysis
Response & completion rates	Response 103/287 (35.9%); complete 101/287 (35.2%); complete/response 101/103 (98.1%).	Results; Methods: CHERRIES reporting
Analysis	Descriptive statistics performed in Microsoft Excel; prespecified coding and sensitivity checks as described.	Methods: Data management and analysis
Tracking & incentives	No email open-rate tracking, IP/cookie checks, or incentives were used.	Methods: CHERRIES reporting

Appendix 2. Survey Instrument (Final, As Administered)

Demographics and Fellowship Training of Residency Leadership in Emergency Medicine (10-Year Follow-Up)

This appendix reproduces the survey instrument as administered to programs. Wording and response options are preserved; layout has been standardized for publication.

Instructions

One response per program (Program Director preferred). If a role is unfilled, select “Not applicable.” No names or personal identifiers were collected.

Section 1. Program Information

1. What is the name of your residency program? (Drop-down list)

2. How many Associate Program Directors does your program have?

3. How many Assistant Program Directors does your program have?

Section 2. Leadership Team (Role-Based Responses)

Please answer the following questions based on how many Associate and Assistant Program Directors there are in your program.

4. Please identify the sex which your program leadership most identifies with.

Response options: Female; Male; Nonbinary; Prefer not to answer.

Roles to complete: Program Director; Associate Program Director 1; Associate Program Director 2; Assistant Program Director 1; Assistant Program Director 2; Assistant Program Director 3; Assistant Program Director 4.

Combined Length at a Residency Leadership Position

Response options: <5 years; 5–10 years; >10 years.

Roles to complete: Program Director; Associate Program Director 1; Associate Program Director 2; Assistant Program Director 1; Assistant Program Director 2; Assistant Program Director 3; Assistant Program Director 4.

Fellowship?

Response options: None; Administration; Critical Care; Medical Education; EMS; Simulation; PEM; Ultrasound; Toxicology; Other.

Roles to complete: Program Director; Associate Program Director 1; Associate Program Director 2; Assistant Program Director 1; Assistant Program Director 2; Assistant Program Director 3; Assistant Program Director 4.

Secondary Board Certification?

Response options: None; Critical Care; Internal Medicine; Pediatrics; Surgery; Other.

Roles to complete: Program Director; Associate Program Director 1; Associate Program Director 2; Assistant Program Director 1; Assistant Program Director 2; Assistant Program Director 3; Assistant Program Director 4.

Open-ended comments (optional): ____
